# Pressure‐Tuneable Visible‐Range Band Gap in the Ionic Spinel Tin Nitride

**DOI:** 10.1002/anie.201805038

**Published:** 2018-08-08

**Authors:** John S. C. Kearney, Miglė Graužinytė, Dean Smith, Daniel Sneed, Christian Childs, Jasmine Hinton, Changyong Park, Jesse S. Smith, Eunja Kim, Samuel D. S. Fitch, Andrew L. Hector, Chris J. Pickard, José A. Flores‐Livas, Ashkan Salamat

**Affiliations:** ^1^ Department of Physics and Astronomy, and HiPSEC University of Nevada, Las Vegas Las Vegas NV 89154 USA; ^2^ Department of Physics Universität Basel 4056 Basel Switzerland; ^3^ High Pressure Collaborative Access Team Geophysical Laboratory, Carnegie Institute of Washington Argonne IL 60439 USA; ^4^ Chemistry University of Southampton Southampton SO17 1BJ UK; ^5^ Department of Materials Science and Metallurgy University of Cambridge Cambridge CB3 0FS UK; ^6^ Advanced Institute for Materials Research Tohoku University Sendai 930-8577 Japan

**Keywords:** ab initio calculations, high-pressure chemistry, nitrides, semiconductors

## Abstract

The application of pressure allows systematic tuning of the charge density of a material cleanly, that is, without changes to the chemical composition via dopants, and exploratory high‐pressure experiments can inform the design of bulk syntheses of materials that benefit from their properties under compression. The electronic and structural response of semiconducting tin nitride Sn_3_N_4_ under compression is now reported. A continuous opening of the optical band gap was observed from 1.3 eV to 3.0 eV over a range of 100 GPa, a 540 nm blue‐shift spanning the entire visible spectrum. The pressure‐mediated band gap opening is general to this material across numerous high‐density polymorphs, implicating the predominant ionic bonding in the material as the cause. The rate of decompression to ambient conditions permits access to recoverable metastable states with varying band gaps energies, opening the possibility of pressure‐tuneable electronic properties for future applications.

At ambient conditions, a vastly explored path to tuning electronic properties is the use of dopants, secondary components that influence the electronic density of states near the Fermi level. Beyond some critical concentration, however, dopants can cause irreversible changes to the structure and chemistry of the host, limiting the range of applicability. In Sn binaries, N‐doped SnO_2_ has been successfully demonstrated, with distinct changes in the electronic and structural properties.^1^, ^2^ Within its ambient rutile structure, replacing up to 60 % of O sites with N sees the band gap close by 0.3 eV as O 2p in the upper valence band are replaced with higher‐energy N 2p electrons,^1^ and complete substitution sees formation of the Sn_3_N_4_ with the spinel (*Fd* $\bar{3}$ *m*) structure.

The Group 14 elements, with the exception of Pb, form nitrogen‐rich nitrides with *X*_3_N_4_ stoichiometry with a range of differing, interesting physical properties.^3^ Various dense C_3_N_4_ materials have been reported with varying carbon hybridisation,^4^, ^5^ and Si_3_N_4_ and Ge_3_N_4_ each have several high‐pressure phases, including a cubic spinel (*Fd* $\bar{3}$ *m*) structure.^6^ Sn_3_N_4_ was discovered in 1909,^7^ and its first bulk preparation was described by Maya in 1991 by the thermal decomposition of an amide–imide polymer derived from SnBr_4_ and KNH_4_.^8^ The spinel crystal structure was then determined by Scotti in 1999 at ambient conditions.^9^ Preparation by high pressure metathesis reactions,^10^ a urea‐gel route,^11^ and various thin‐film methods^12^, ^13^, ^14^, ^15^ have also been described. Sn_3_N_4_ has a tunable band gap, and is composed of only Earth‐abundant elements, identifying it as a favourable candidate for optoelectronic technologies.^16^ It has also been investigated as a photocatalyst for water oxidation,^16^, ^17^ a selective sensor for ethanol,^11^ and a cathode material in Li and Na batteries.^18^

Pressure is a powerful probe for useful electronic and structural phenomena. Intuitively, volume reduction in non‐metals increases overlap between neighbouring orbitals, and increases band dispersion, reducing the conduction band minimum (CBM) and increasing the valence band maximum (VBM). The expected response to pressure is therefore a closure of the band gap, and eventual metallisation. This has spurred significant research in recent decades, and is a promising route to room‐temperature superconductivity.^19^, ^20^, ^21^, ^22^ The inverse behaviour, band gaps opening with pressure, has recently been a subject of interest with discontinuous gap opening in alkali metals^23^ upon forming electride structures at extreme compression,^24^ and continuous gap opening has long been known for covalent semiconductors.^25^ Herein we report Sn_3_N_4_, a unique ionic semiconducting material with a spinel structure that is stable over a range of 80 GPa, and which exhibits a reversible band gap opening of about 2 eV. Furthermore, two high‐density phases (*P*2_1_/*c* and *R* $\bar{3}$ *c*) are experimentally confirmed by thermal annealing at 56 and above 105 GPa, respectively. Both phases are predicted to exhibit pressure‐mediated band gap opening.

Sn_3_N_4_ was produced in the spinel phase from the reaction of SnCl_4_ and LiNH_2_ at 610 K and 50 atm. Elemental analysis confirms no oxygen contamination in our starting material, which has been reported by others (up to 5 % replacement of N with O) using various deposition techniques.^16^, ^26^, ^27^ The resulting nanocrystalline powder is brown in colour and opaque to visible light (Supporting Information, Figure S1), its optical band gap measured by the onset of absorption is about 1.3 eV. While our measured value differs from reported thin‐film values,^16^, ^28^ it is difficult to draw a direct comparison when the composition is different and contamination is present. Meanwhile, our value lies close to our band gap calculated with a single‐shot Green's function (*G*_0_ *W*_0_) approach −1.4 eV and is within the range of prior values reported from computational methods: 1.1–1.55 eV from density functional theory (DFT), modified Becke–Johnson, and single‐shot Green's function (*G*_0_ *W*_0_) approaches.^16^, ^28^, ^29^, ^30^

On compression in a diamond anvil cell at room temperature, Sn_3_N_4_ first becomes red and ultimately transparent to visible light (Figure 1 a). Measurements of the optical absorption (Figure 1 b) reveal that the increase in transparency corresponds to a continuous blue‐shift of the optical gap as a function of pressure at a rate of about 17 meV GPa^−1^, reaching a value of 3.0 eV at pressures of about 100 GPa (Figure 1 c). This electronic response is reversible, and features hysteresis, which is often thought to aid the stabilisation of high‐pressure or high‐temperature characteristics to ambient conditions.^1^, ^31^ For instance, the band gap at ambient conditions is highly dependent on the rate of decompression (green square in Figure 1 c, and Supporting Information, Figure S3) and the presence of temperature annealing (green circle in Figure 1 c).

**Figure 1:**
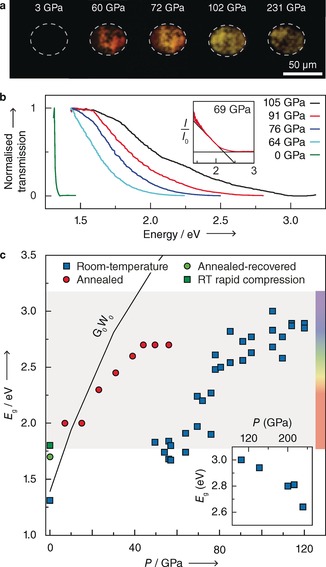
a) In situ rear‐illuminated photomicrographs taken during compression showing an increase in optical transmission to 102 GPa, followed reduced transmission up to 231 GPa. b) Selected optical absorption spectra showing blue‐shift in absorption edge with pressure. Inset: Procedure for deriving the optical band gap from absorption spectra. c) Measured and calculated optical band gap for spinel (*Fd* $\bar{3}$ *m*) Sn_3_N_4_ with pressure. All data collected with no PTM unless specified. Blue squares: room‐temperature compression; red circles: ohmically annealed at 578 K, and sample allowed to cool before measurement; green square: recovered from 80 GPa with rapid compression (Ne PTM); green circle: recovered from 50 GPa with sample annealed at 578 K during compression; black line: band gap calculated with *G*_0_ *W*_0_.

Independent of computational method (DFT with PBE0 hybrid functional or *G*_0_ *W*_0_), our calculations show an opening of the optical gap with pressure. Optical absorption measurements were intentionally performed without pressure transmitting media (PTM) to avoid false radiation (Figure 1 c, blue squares). To overcome the non‐ideal response owing to anisotropic strain and minimise stress at grain boundaries without the use of a PTM, we ohmically annealed the samples to 578 K, allowing samples to cool to room temperature before measuring the optical absorption edge (Figure 1 c, red circles). This method of annealing reveals a band gap opening response aligned with our *G*_0_ *W*_0_ calculations, most likely by reducing artificial gap closure in the recorded signal owing to absorption at anisotropically stressed grain boundaries.

Experimentally, there is a turning point in the band gap evolution beyond 100 GPa, and the optical gap reduces to a value of 2.64 eV by 231 GPa (rightmost photograph in Figure 1 a, and inset of c). Band gap closure is not predicted by calculations on *Fd* $\bar{3}$ *m* and, accordingly, the presence of new phases emergent under compression was considered for the Sn_3_N_4_ composition by ab initio random structure searching (AIRSS).^32^, ^33^ DFT calculations with the PBE0 hybrid functional on the enthalpically low‐lying predicted structures reveal a monoclinic (*P*2_1_/*c*) structure which becomes favourable compared to *Fd* $\bar{3}$ *m* at 40 GPa (Figure 2 a). The *P*2_1_/*c* phase is replaced by a rhombohedral (*R* $\bar{3}$ *c*) structure commencing at 87 GPa, and this phase occupies a narrow pressure range before it is replaced by a cubic (*I* $\bar{4}$ 3*d*) structure at 97 GPa (Figure 2 b).

**Figure 2:**
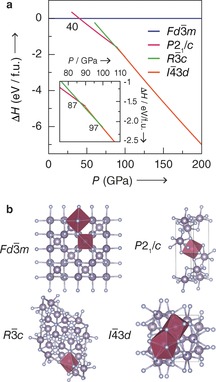
a) DFT‐calculated enthalpies as function of pressure for four phases of Sn_3_N_4_, relative to the ambient spinel (*Fd* $\bar{3}$ *m*) structure. b) Unit cell diagrams of Sn_3_N_4_ in the spinel phase and three predicted high‐density polymorphs, *P*2_1_/*c*, *R* $\bar{3}$ *c*, and *I* $\bar{4}$ 3*d*. Sn purple, N pale purple; Sn coordination polyhedra are highlighted for each structure.

In synchrotron X‐ray diffraction (XRD) experiments, the predicted high‐density phases are not observed under non‐hydrostatic (no PTM), or quasi‐hydrostatic compression in a Ne PTM. Rather, the *Fd* $\bar{3}$ *m* structure persists to at least 80 GPa in the quasi‐hydrostatic run, after which it was not possible to index as a cubic cell, which is most likely due to anisotropic strain of the sample environment. Within the *Fd* $\bar{3}$ *m* phase, we record the volume (Figure 3 a) and fit a third‐order Birch–Murnaghan equation of state to derive the bulk modulus *K*_0_=191.8(5) GPa and pressure‐derivative *K*_0_′=3.97(3). Our recorded value is 28 % larger than previous reports,^34^ but 5 % smaller than our DFT‐derived compressibility (*K*_0_=201.4, *K*_0_′=4.37, blue dashed line in Figure 4). In the non‐hydrostatic run, deviatoric stresses meant that the *Fd* $\bar{3}$ *m* phase was no longer possible to index as cubic as low as 25 GPa, although the diffraction features of the phase remained prominent and there was no evidence of a structural transition up to the highest measured pressure of 50 GPa. Fitting these volume‐pressure data gives a bulk modulus *K*_0_=237(4) GPa and *K*_0_′=3.6(2). This difference in measured compressibilitiy of the *Fd* $\bar{3}$ *m* phase compared with quasi‐hydrostatic conditions is a consequence of additional inhomogeneous strain. When no PTM is employed, the surface and strain energy of the system can raise much more quickly, lowering to phase transition pressures and even allowing some material to undergo pressure‐induced amorphisation.^35^

**Figure 3:**
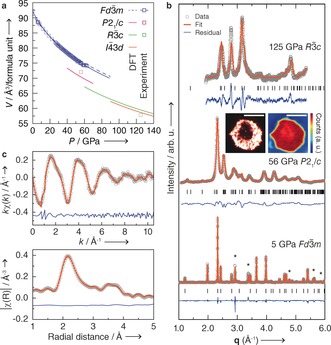
a) Volume–pressure relationship per formula unit of the four predicted and three confirmed crystal phases of Sn_3_N_4_. b) X‐ray diffraction patterns of spinel Sn_3_N_4_ and two high pressure phases *P*21/*c* and *R* $\bar{3}$ *c* at 5, 56, and 125 GPa, respectively. *Fd* $\bar{3}$ *m* and *P*2_1_/*c* were subject to Rietveld refinements and *R* $\bar{3}$ *c* to Le Bail refinements. Black tick marks (|) denote expected Bragg reflections from each Sn_3_N_4_ phase and asterisks (*) from the Ne pressure medium where applicable (the *P*2_1_ *c* and *R* $\bar{3}$ *c* phase were accessed used ohmically heating and no PTM). Insets: images of sample environment (pure Sn_3_N_4_) following thermal annealing at 56 GPa from a visible light microscope (left) and imaged by X‐ray transmission (right), showing regions with distinctly different optical band gaps and densities. Scale bars: all 50 μm. c) Structure fitting to 105 GPa EXAFS spectrum using the *R* $\bar{3}$ *c* structure type, after using CO_2_ laser heating to overcome kinetic barriers.

**Figure 4:**
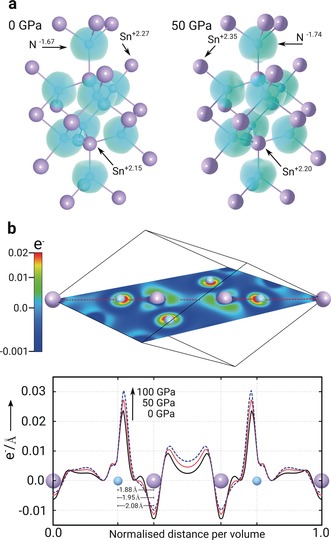
a) Electron localisation function (ELF) (characteristic electron‐gas value of 0.5) for *Fd* $\bar{3}$ *m* Sn_3_N_4_ showing the ionic character of the bonding. Under pressure, electronic charges become more localised around the atoms (depicted Bader charges). In all model structures, Sn atoms are depicted in purple and N in blue. b) Conduction band charge density for *Fd* $\bar{3}$ *m* at ambient pressure along the (0,−1,1) lattice plane (colour bar indicates the electron charge per volume (Å^−3^), and (below) projected along the red path at 0, 50, and 100 GPa normalised per volume.

To realise the predicted high‐pressure phases, it was thus necessary to anneal with temperature. Annealing at 578 K for 15 minutes at 56 GPa yielded a phase‐separated sample comprising *Fd* $\bar{3}$ *m* at its periphery, and *P*2_1_/*c* at its centre (Figure 3 b insets). The striking difference in transparency follows our calculations, which predict closure of the band gap by 0.38 eV across this transition (Supporting Information, Figure S4). However, while computation predicts and annealing demonstrates a discontinuous closure of the band gap across the first‐order transitions into the *P*2_1_/*c* and the *R* $\bar{3}$ *c* phases, pressure‐mediated band gap opening is present in each of the structures. As such, a likely reason for the turning point in the optical band gap above 100 GPa is not a crystalline phase change, but rather a pressure‐induced amorphisation owing to anisotropic strain,^36^ which is a consequence of the large energetic difference between *Fd* $\bar{3}$ *m* and the ground state at these pressures, reinforcing the need for thermal annealing.

XRD collected at the dark centre of the annealed sample can be modelled well with the predicted *P*2_1_/*c* structure by Rietveld analysis (Figure 3 b). A sample annealed to 800 K at 125 GPa can be modeled by the *R* $\bar{3}$ *c* structure with Le Bail analysis, and further information was sought via X‐ray absorption spectroscopy (XAS). While XRD signal strength varies with atomic number, reducing information gained on smaller atoms when in the presence of heavier nuclei, XAS (particularly extended X‐ray absorption fine structure, EXAFS) probes nearest‐neighbour distances, allowing the locations of N atoms to be refined (Figure 3 c), and complete crystallographic information to be extracted (Supporting Information, Table S11). EXAFS measurements were performed on samples which were CO_2_ laser annealed with in situ XRD confirming the complete and homogeneous structural change.^37^

Note that while the calculated ground state structure above 97 GPa is *I* $\bar{4}$ 3*d*, we experimentally observe *R* $\bar{3}$ *c* up to 125 GPa. Kinetic barriers were not considered and most likely conditions that permit access to *I* $\bar{4}$ 3*d* were not met.^38^ Structurally, the two phases are difficult to distinguish using XRD, but a clear difference is the local environment of the Sn atoms: *R* $\bar{3}$ *c* has 7 N in its first coordination shell, and *I* $\bar{4}$ 3*d* has 8. Further, the ordering of those N atoms differs greatly, with *R* $\bar{3}$ *c* showing greater static disorder in its first shell than *I* $\bar{4}$ 3*d*. EXAFS measurements, which are sensitive to local environment, thus allow us to clearly distinguish between these different Sn sites, conclusively showing *R* $\bar{3}$ *c* to be the phase emergent upon annealing at 125 GPa.

Past examples of pressure‐induced band gap opening are well‐documented in tetravalent semiconductors, wherein the magnitude of the shift diminishes with polarity of the bonding (that is, from IV to II–VI).^25^ In the zinc chalcogenides, pressure increases the band gap across the 3–4 eV range in cubic ZnS and 2 forms (Wurtzite and rock salt) of ZnO.^39^, ^40^ CdTe exhibits a band gap opening which lies close to the visible spectrum within its zincblende and cinnabar structures,^41^ but quickly undergoes a transition to a rock salt structure above about 4 GPa, which is accompanied by abrupt closure of the gap to a near‐metallic state. Prior to observations made here, only the III–V GaAs has shown pressure‐mediated band gap opening across the visible range, within its zinc blende structure up to 17 GPa,^42^ where it transforms into an orthorhombic phase with significant band‐gap closure. Within tetravalent semiconductors, transitions to metallic or small‐gap structures are thus prevalent. In contrast, structural transitions in Sn_3_N_4_ up to 125 GPa are accompanied by a comparatively small band‐gap closure, and each of the observed phases exhibit gap opening with pressure (Supporting Information, Figure S4) This is the case for other Sn^IV^ compounds: SnO_2_ has been predicted to exhibit pressure‐mediated band gap opening in the 3–5 eV range across numerous phases,^43^ and has been measured within its rutile structure.^44^

To form Sn_3_N_4_, Sn donates its 5s and 5p electrons to fill the unoccupied 2p levels in N. The resulting compound has a flat valence band comprising primarily N‐p electrons, showing only small levels of hybridisation with Sn‐d states. In contrast, the conduction band is disperse, and formed primarily by Sn‐s states with contributions from N‐s states (Supporting Information, Figure S7). Electron localisation function (ELF) analysis of the spinel phase (Figure 4 a) shows distinct localisation of valence electrons around the N atoms, demonstrating the ionic character of the bonding, and almost no visible change in the ELF spheres under pressure evidences its robustness. In fact, Bader charges under pressure (Figure 4 a and Supporting Information, Figure S8) show an increase in polarisation for both Sn and N as the volume decreases, equating to greater ionic exchange.

Investigating conduction band charge density across the spinel unit cell (Figure 4 b) gives further insight into the mechanism behind the band gap opening. A decrease in the interatomic spacing by 0.2 Å over 100 GPa is accompanied by an increase in localisation of charge density, notably around N atoms and in the interstitial sites between Sn atoms. The increased localisation with pressure produces deeper potentials, making unoccupied levels in the CBM harder to access energetically. While the same effect is observed in the VBM, the p and d character of those states results in a much smaller shift, and the net effect is a growth of the band gap. Both the ionic bonding and localisation effect are observed for *Fd* $\bar{3}$ *m*, *P*2_1_/*c*, and *R* $\bar{3}$ *c* phases, with the pressure shift of the band gap lessening in the high‐density phases (Supporting Information, Figures S9–S11). Again, this is analogous to tetravalent semiconductors, where the VBM comprises p bonding states and the CBM s states localised around the nuclei,^25^ but while their fate is typically a transition into a narrow‐gap or metallic state due to significant orbital overlap, the lack of hybridisation in Sn_3_N_4_ creates stability over a large pressure range, as well as having pressure‐mediated band gap opening a common phenomenon across several high‐pressure phases.

Highly stable materials are usually wide‐gap insulators, where covalency dominates the ionic exchange, such as diamond,^45^ MgO,^46^ and LiH,^47^ whereas the enhanced stability of Sn_3_N_4_ to applied pressure and temperature can be attributed to its dominant ionic character. We demonstrate control of structure via selective thermodynamic conditions and the tunability of the band gap across the entire visible range, an insight into future chemical doping. The dependency of recovered states on decompression pathways and rates suggests tunability to desired electronic gaps. Large‐scale samples may be accessed via large‐volume static or shock‐recovered dynamic techniques. Sn_3_N_4_ is the first ionic semiconductor demonstrated to have such stability and technologically‐useful electronic response. The mechanism governing pressure‐mediated band gap opening is solely due to the nature of the bonding, and our preliminary calculations on spinel Ge_3_N_4_ and Si_3_N_4_ as well as previous data^29^ suggest a similar pressure‐mediated band gap opening. Such chemistry can be sought in similar systems, potentially defining a new class of simple ionic semiconductor materials.

## Conflict of interest

The authors declare no conflict of interest.

## Supporting information

As a service to our authors and readers, this journal provides supporting information supplied by the authors. Such materials are peer reviewed and may be re‐organized for online delivery, but are not copy‐edited or typeset. Technical support issues arising from supporting information (other than missing files) should be addressed to the authors.

Supplementary
