# Supplementary material for: Pressure‐Tuneable Visible‐Range Band Gap in the Ionic Spinel Tin Nitride
Source: Angew Chem Int Ed Engl. 2018 Aug 8;57(36):11623–8. doi: 10.1002/anie.201805038 (PMC6221123; doi:10.1002/anie.201805038)
Supplement: Supplementary file 1 — Supplementary [file ANIE-57-11623-s001.pdf]

Angewandte Chemie
Eine Zeitschrift der Gesellschaft Deutscher Chemiker

Supporting Information

Pressure-Tuneable Visible-Range Band Gap in the Ionic Spinel Tin Nitride

John S. C. Kearney, Miglė Graužinytė, Dean Smith, Daniel Sneed, Christian Childs, Jasmine Hinton, Changyong Park, Jesse S. Smith, Eunja Kim, Samuel D. S. Fitch, Andrew L. Hector, Chris J. Pickard, José A. Flores-Livas,* and Ashkan Salamat**

anie_201805038_sm_miscellaneous_information.pdf

Supplementary Information

1 Methods

1.1 High pressure experiments

Samples were loaded quasi-hydrostatically in a pressure-transmitting medium (PTM) of Ne for synchrotron X-ray experiments, in KBr for laser heating, and loaded with no PTM for optical band gap and ohmic heating measurements. Rhenium foil was pre-indented and drilled by electronic discharge or laser micromachining [1] to form a gasket for all experiments except for X-ray absorption spectroscopy (XAS), where beryllium is substituted. Pressure measurements were calibrated with the shift of the fluorescence lines of ruby [2] or the shift in the first-order Raman frequency of the diamond tip. [3]. No detection of the O_2 vibron or SnO/SnO_2 Raman peaks were observed before or after heating.

1.2 CO_2 laser and ohmic annealing

A Synrad 125 W CO_2 laser was used to heat the samples at 50% power for no longer than 30 seconds. [4, 5] An ohmic heater constructed in-house was used to anneal the samples in a DAC under pressure. The circular heater was placed around the gasket in the DAC and a thermocouple attached to one of the diamonds was used for temperature measurements. Ohmic heating was performed at 56 and 125 GPa from room temperature to 578 K and 800 K, respectively, in increments of 25 K, and heating for 30 minutes at each temperature increment.

1.3 Optical band gap experiments

Measurements of optical transmission were carried out using light from a 500 W tungsten bulb, focused through the sample environment, and subsequently analysed with either an Ocean Optics HR2000+ES spectrometer sensitive from 190-1100 nm - allowing for optical band gap measurements from 1.20-3.25 eV, or an InGaAs photodiode sensitive from 2400-1000 nm (0.6-1 eV), using a tuneable bandpass filter for bandwidth selection. Both methods have an uncertainty of 0.2 eV. False transmission was avoided by loading the Sn_3N_4 sample in the absence of a pressurising medium. Measurements were taken from

two non-hydrostatic DAC experiments up to 230 GPa without heating. Additional measurements were collected in two non-hydrostatic loadings at fixed pressures of 55 and 95 GPa, thermally annealed by resistive heating at 578 K and 800 K respectively.

1.4 Synchrotron X-ray diffraction experiments

Angle-dispersive X-ray diffraction patterns were collected at the beamlines of 16-ID-B and 16-BM-D of HPCAT, Advanced Photon Source with X-ray photon energies of 30.5 keV (0.4066 Å) or 25 keV (0.4959 Å) respectively. The two-dimensional images were integrated using the Dioptas software package. [6]

1.5 X-ray absorption spectroscopy

Near-edge (XANES) and extended fine-structure (EXAFS) absorption measurements were taken at the 16-BM-D beamline of HPCAT to probe the Sn K-edge at 29.2 keV. Data was taken at a range of X-ray photon energies from 28.8 keV to 30.0 keV. XAS data was collected at RT for 23 pressure points from ambient to 64 GPa in a quasi-hydrostatic loading with neon PTM. XAS data of laser-annealed samples at 55 GPa and 105 GPa were also collected where KBr was used as a PTM. A CO_2 laser was used for annealing at UNLV and in situ on the 16-ID-B beamline at APS.

1.6 Computational methods

Searches on the enthalpy landscape were conducted with AIRSS [7, 8] for cells of different formula units of Sn_3N_4 and for different pressures up to 200 GPa. Energy, atomic forces, and stresses were evaluated at the hybrid-DFT level using the PBE0 functional. [9] A plane-wave basis set with a cut-off of 950 eV and $4{\times}4{\times}4$ Γ-centered mesh were used in the projector augmented wave (PAW) method as implemented in the Vienna Ab Initio Simulation Package (VASP). [10] Geometry relaxations were halted as soon as forces and stresses reached the thresholds of 2 meV $\mathring{A}^{-3}$ and 0.1 meV $\mathring{A}^{-3}$, respectively. Obtained structures were used to calculate the quasi-particle band gaps employing the G0W0 approach. These many-body calculations were carried out employing the linearized augmented plane-wave + local orbitals (LAPW+lo) basis set as implemented in the all-electron full-potential code "exciting". [11, 12, 13] The quasi-particle correction was applied to Kohn-Sham energies obtained within the local-density approximation using the LAPW cut-off $R_{\mathrm{MT}}\,|\mathbf{G}+\mathbf{k}|_{\mathrm{max}} = 9$ and $8 \times 8 \times 8$ Brillouin zone sampling. The dielectric function was computed on a $2 \times 2 \times 2$ Γ-centered **k**-grid using 600 empty states and 32 frequencies. These settings ensure that the quasi-particle band gaps are converged to 0.1 eV or better.

2 Ambient Sn_3N_4

Sn_3N_4 was prepared under solvothermal conditions in an inconel autoclave (Parr 4740CH) with a silica liner. Under N_2 0.6 g $SnCl_4$ and 30 cm^3 benzene were placed in the liner, then 0.211 g $LiNH_2$ was added to the solution and stirred. The autoclave was sealed and heated to 610 K for 12 hours. Typically 50 Atm pressure developed during heating. After cooling to room temperature, the solid was washed with deionised water (100 ml) and MeOH (50 ml) to remove the LiCl by-product. The powder was further washed with 3 M HCl (50 ml) to remove a tin metal contaminant due to thermal decomposition of the nitride. Combustion (CHN) analysis was outsourced to Medac Ltd with samples decomposed with a WO_3 combustion aid to maximise nitrogen recovery. The sample contained 11.5% nitrogen (13.58% calcd. for Sn_3N_4), as well as 5.6% carbon and 1.5% hydrogen, may be attributed to the decomposition of benzene. [14]

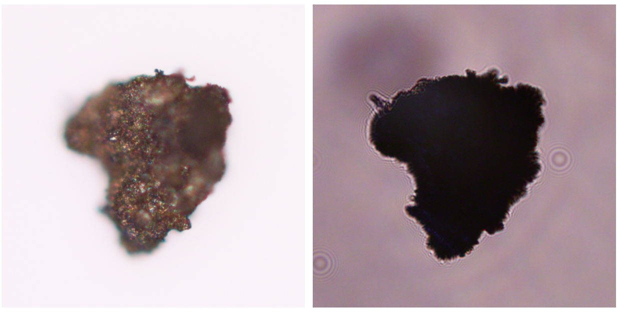

Figure S 1: Micrographs of ambient starting Sn_3N_4 sample. (left) In reflection lighting geometry. (right) In transmission lighting geometry

3 Annealed high-pressure phases

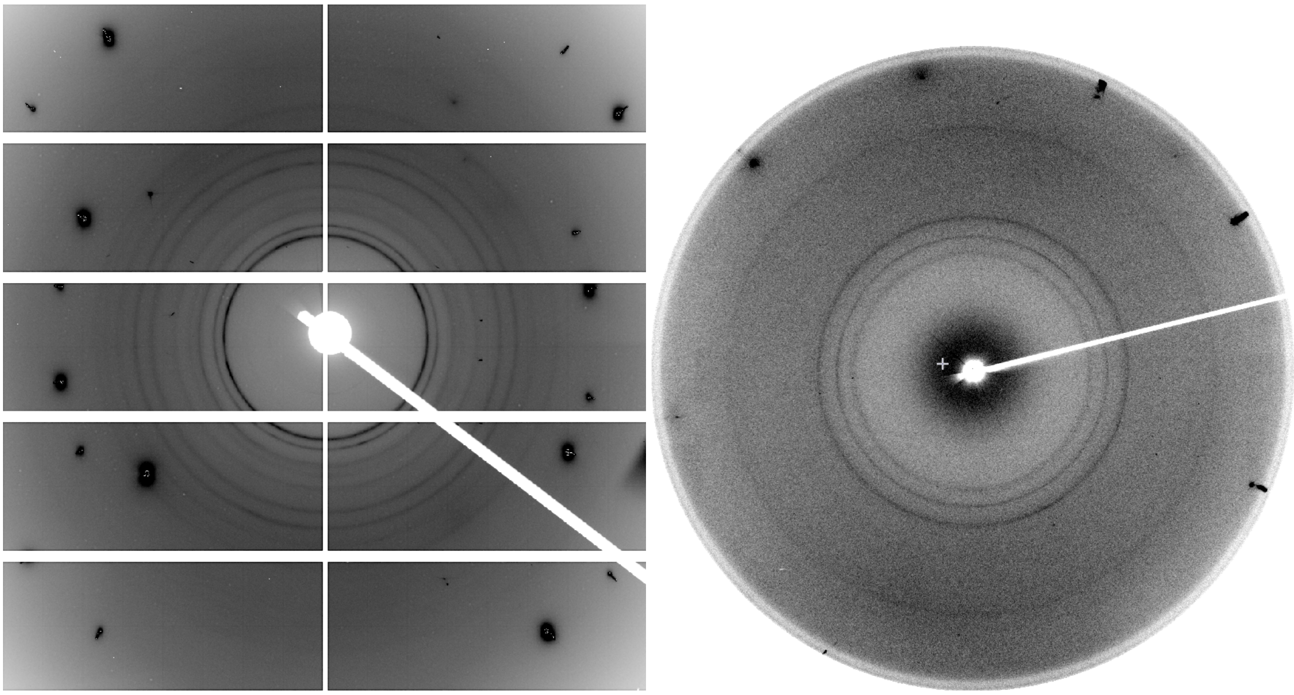

Figure S 2: Powder diffraction of Sn_3N_4 in two high-pressure polymorphs following thermal annealing. (left) $P2_1/c$ following annealing at 578 K at 58 GPa (right) $R\overline{3}c$ following annealing at 800 K at 125 GPa.

4 Recovered metastable states

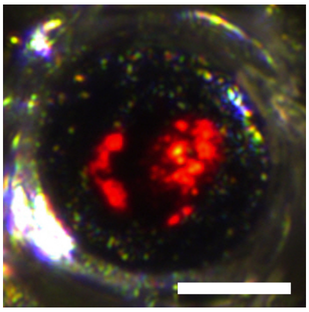

Figure S 3: Recovered spinel Sn_3N_4 at ambient pressure, inside a DAC, following rapid compression to 80 GPa and subsequent rapid decompression. The sample maintains a band gap which is greater than its ambient 1.3 eV, and within the red region of visible spectrum ($\sim$ 1.8 eV). Scale bar 100 μm.

5 Band gap predictions

Figure 4 summarizes the calculated Γ-Γ (optical) band gap for different polymorph of Sn_3N_4 as a function of pressure. Dashed lines shows the hybrid-DFT (PBE0) calculations and solid lines are G_0W_0 values. Obtained structures were used to calculate the quasi-particle band gaps employing the G_0W_0 approach. These many-body calculations were carried out employing the linearized augmented plane-wave + local orbitals (LAPW+lo) basis set as implemented in the all-electron full-potential code `exciting` [11, 12, 13]. The quasi-particle correction was applied to Kohn-Sham energies obtained within the local-density approximation using the LAPW cutoff $R_{\mathrm{MT}}\,|\mathbf{G}+\mathbf{k}|_{\mathrm{max}} = 9$ and the $8 \times 8 \times 8$ Brillouin zone sampling. The dielectric function was computed on the $2 \times 2 \times 2$ Γ-centered **k**-grid using 600 empty states and 32 frequencies. These settings ensure that the quasi-particle band gaps are converged up to 0.1 eV or better.

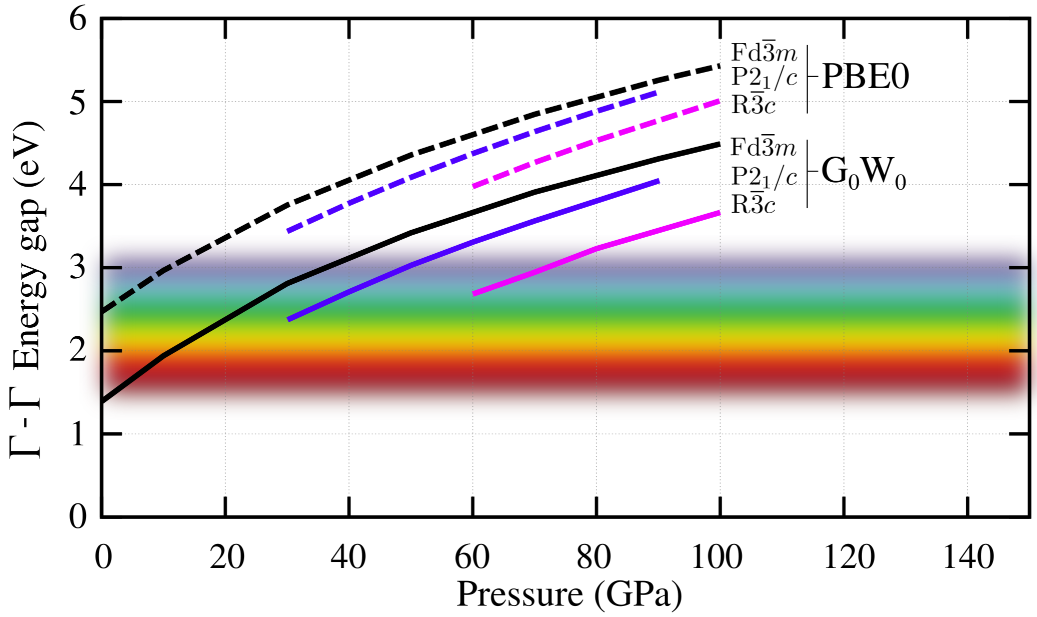

Figure S 4: **Calculated band gaps for several polymorphs of Sn_3N_4 under pressure.** Dashed lines show hybrid PBE0 and solid lines G_0W_0 calculations.

6 Methodology for EXAFS analysis

All data handling was carried out using the DEMETER software package developed by Bruce Ravel. ATHENA was used for data processing, calibration, and file merging. A tin foil standard was used for calibrating the tin K-edge position of 29200 eV. For data analysis, the ARTEMIS package was used as the front end for *ab initio* calculations of scattering amplitudes using muffin tin potentials *via* Feff, as well as fitting *via* the FEFFIT fitting code.

6.0.1 105 GPa data set

For data analysis, the Artemis package was used as the front end for Feff calculations and Feffit fitting. Using ab initio calculations, the ground state structure of Sn_3N_4 at 105 GPa has been predicted to be either the trigonal structure with the $R\bar{3}c$ space group, or the cubic structure in the $I\bar{4}3d$ space group. Crystallographically, the two structure types show very similar diffraction patterns, making it very difficult to solve the structure. One major difference between the two structure types is the local coordination of the tin atoms. The $R\bar{3}c$ structure has 7 nitrogen atoms in the local environment of the tin, whereas the $I\bar{4}3d$ phase structure has 8 nitrogen atoms. Due to its sensitivity to the local coordination of the absorbing atom, EXAFS is a useful tool for verifying the structure type. Each structure type was tested against the 105 GPa data set. Feff input files were generated from the crystallographic information files created from the predicted structures. For fitting, the following fitting model was used for both structures:

1. The Correlated Debye model can be used to describe the disorder of the tin atoms.

2. The Einstein model can be used describe the disorder of the nitrogen atoms.

3. Shell displacements are independent.

4. Shell displacements for the Nitrogen and tin atoms are independent.

5. The Fermi energy correction of the first shell is independent of the rest of the shells.

Fitting results for the $R\bar{3}c$ structure type:
Using the Nyquist criterion for the total number of independent points in a packed signal, there were 36.33 independent points in this data set. Of those points, 22 variables were required to adequately fit to the EXAFS spectrum. The following table lists the guess parameters used:

The parameter for Δ R of each nitrogen shell and each tin shell was given an independent variable. As stated earlier, the disorder of the tin atoms was described using the Debye model, and the Debye temperature for this structure is given by the parameter thetad, this can be correlated to the Debye-Waller terms for each of the tin atoms, and these values are given in table S 10. As in the ambient data, do to the large mass of tin, the core-hole broadening effects was considered, in order to properly account for this, the electron self energy term, Ei, was included for the tin sites. This was given by the parameter EiSn, which was evaluated to 8.41 +/- 1.48 eV. The Einstein model was used to describe the nitrogen disorder, and the Einstein temperature was given the parameter thetaeN, using this, it was determined that the Debye-Waller term for the nitrogen atoms was 0.001 $Å^2$. Two Fermi energy

Table S 1: A table of guess parameters and the corresponding fit values for the EXAFS modeling of the $R\bar{3}c$ structure

Guess parameters	Fit values
amp	0.94479809 +/- 0.03481503
enot	4.20051391 +/- 0.57826025
ShiftN1	-0.07410339 +/- 0.004779
shiftN3	-0.04897973 +/- 0.03381823
shiftN4	0.24026609 +/- 0.02431603
ShiftSn1	0.0161526 +/- 0.01539959
thetad	262.9198407 +/- 51.89840894
c3N	-0.00359008 +/- 0.00052573
c4N	0.0002104 +/- 0.00003867
shiftSn2	0.14278195 +/- 0.10100948
thetaeN	1508.14934 +/- 782.0977546
EiSn	8.41935328 +/- 1.48347466
shiftSn3	0.08853489 +/- 0.11676499
enot1	2.3835039 +/- 0.38692296
Set parameters	
temp	300
mmc	0.00012

corrections were used, one for the first coordination shell (Enot1), and one for the rest (Enot). This compensates for incomplete core-hole shielding between the nitrogen and the tin in the first coordination shell. The McMaster correction given from the Feff calculation was added to all disorder terms through the set parameter mmc. The following correlations between fit parameters were above 80%:

Table S 2: Correlation between fit parameters for $R\bar{3}c$ structure

Correlations between variables	
enot1 & shiftn1	0.8761
thetaen & amp	-0.8615

Table S 3: Final fit values of atoms out to third coordination shell for the $R\bar{3}c$ structure

Name	N	S02	sigma^2	e0	delr	Reff	R
Nitrogen 2.1	2	0.945	0.00142	2.384	-0.0741	2.0231	1.949
Nitrogen 2.2	2	0.945	0.00142	2.384	-0.0741	2.1254	2.0513
Nitrogen 1.1	2	0.945	0.00142	2.384	-0.0741	2.1786	2.1045
Nitrogen 2.3	1	0.945	0.00142	2.384	0.24027	2.3401	2.58037
Tin 1.1	4	0.945	0.00828	4.201	0.01615	2.9058	2.92195
Tin 1.2	2	0.945	0.00841	4.201	0.01615	3.0119	3.02805
Tin 1.3	2	0.945	0.00843	4.201	0.01615	3.0284	3.04455
Nitrogen 2.4	2	0.945	0.00142	4.201	0.24027	3.0518	3.29207
Nitrogen 2.5	4	0.945	0.00142	4.201	-0.04898	3.7018	3.65282
Nitrogen 1.2	2	0.945	0.00142	4.201	-0.04898	3.9477	3.89872
Nitrogen 2.6	1	0.945	0.00142	4.201	-0.04898	3.9186	3.86962
Tin 1.4	2	0.945	0.00855	4.201	0.01615	3.1486	3.16475
Tin 1.5	4	0.945	0.00888	4.201	0.14278	4.0197	4.16248
Tin 1.6	2	0.945	0.00891	4.201	0.14278	4.1988	4.34158
Tin 1.7	2	0.945	0.00895	4.201	0.14278	4.3572	4.49998
Tin 1.8	4	0.945	0.00914	4.201	0.08854	4.95	5.03854
Tin 1.9	2	0.945	0.00928	4.201	0.08854	5.2482	5.33674
Tin 1.10	2	0.945	0.00928	4.201	0.08854	5.2497	5.33824

Table S 4: Table of electron self energy and cumulant corrections for the fit to $R\bar{3}c$ structure

name	ei	third	fourth
Nitrogen 2.1	0	0	0
Nitrogen 2.2	0	0	0
Nitrogen 1.1	0	0	0
Nitrogen 2.3	0	0	0
Tin 1.1	8.41935	0	0
Tin 1.2	8.41935	0	0
Tin 1.3	8.41935	0	0
Nitrogen 2.4	0	0	0
Nitrogen 2.5	0	-0.00359	0.00021
Nitrogen 1.2	0	-0.00359	0.00021
Nitrogen 2.6	0	-0.00359	0.00021
Tin 1.4	8.41935	0	0
Tin 1.5	8.41935	0	0
Tin 1.6	8.41935	0	0
Tin 1.7	8.41935	0	0
Tin 1.8	8.41935	0	0
Tin 1.9	8.41935	0	0
Tin 1.10	8.41935	0	0

The R-factor by k-weight for this fit is = 1 $\Rightarrow$ 0.00251, 2 $\Rightarrow$ 0.00466, 3 $\Rightarrow$ 0.00892

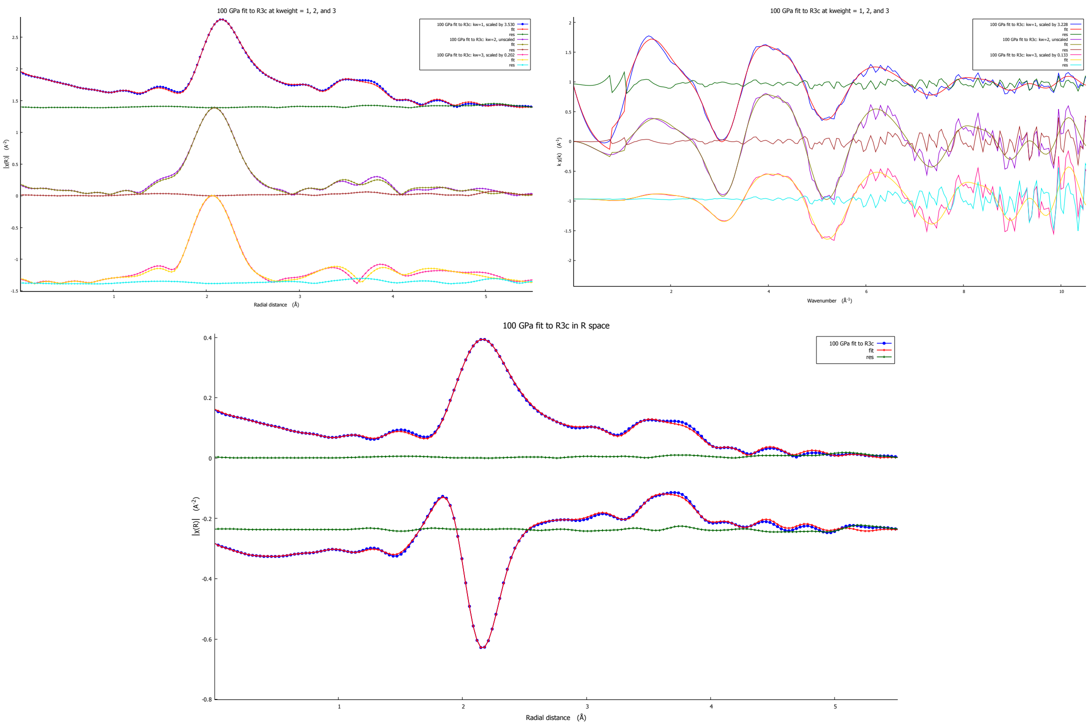

Figure S 5: **Fitting results of 105 GPa data set fit to $R\overline{3}c$ structure.**
Left Top: Fit results to the magnitude of the fourier transform of the EXAFS spectrum weighted by k, k^2, and k^3. **Right Top:** Fit results to the EXAFS spectrum weighted by k, k^2, and k^3. **bottom:** Fit results with residuals to the magnitude of the fourier transform of the EXAFS spectrum and the real part of the fourier transform of the EXAFS spectrum weighted by k. There is a large distortion to the first shell, which in contrast to the previous structures, is a result of the local static disorder of the first coordination shell. In the $R\overline{3}c$ structure, the 7 coordinated nitrogen atoms have 4 unique distances from the absorbing tin atom, creating a large level of static distortion. This fact can also be seen in the high third and fourth cumulants. All spectra are phase corrected using the phase amplitude of the first nitrogen shell.

Fitting results for $I\overline{4}3d$ structure:

Using the Nyquist criterion for the total number of independent points in a packed signal, there were 36.33 independent points in this data set. Of those points, 21 variables were required to properly fit to the EXAFS spectrum. The following tables list the results of the fit:

Table S 5: A table of guess parameters and the corresponding fit values for the EXAFS modeling of the I43d phase of Sn_3N_4

Guess parameters	Fit values
thetae	1732.804765 +/- 18583.16514
ShiftN1	-0.04053558 +/- 0.07234712
Enot	1.56591396 +/- 4.48674995
amp	0.65821211 +/- 0.1277322
ShiftN2	-0.22532116 +/- 0.07308572
thetad	248.7200015 +/- 88.87739861
Ei	5.53845665 +/- 3.91257214
shiftSn2	0.14550924 +/- 0.19475372
shiftSn1	0.0497475 +/- 0.05278369
ShiftN3	2.44897562 +/- 0.516004
shiftSn3	-0.01094084 +/- 0.13143674
shiftSn4	-0.06178016 +/- 1.67850121
C3N	0.02445268 +/- 0.00712705
C4N	0.00030405 +/- 0.00038709
Enot1	2.43839314 +/- 1.10948377
Ei1	14.76184625 +/- 168.7518911
Set parameters	
temp	300
mcm	0.00012

Table S 6: Correlation between fit parameters for the $I\bar{4}3d$ structure

Correlations between variables	
c3n & shift3	0.9792
shift1 & thetae	-0.9585
shift2 & thetae	0.9102
shift2 & shift1	-0.8904

Table S 7: Final fit values of atoms out to third coordination shell for the $I\bar{4}3d$ structure

Name	N	S02	sigma^2	e0	delr	Reff	R
Nitrogen 1.1	4	0.658	0.00124	2.438	-0.04054	2.0194	1.97886
Nitrogen 1.2	4	0.658	0.00124	2.438	-0.22532	2.3265	2.10118
Tin 1.1	8	0.658	0.00926	1.566	0.04975	2.9248	2.97455
Tin 1.2	4	0.658	0.00975	1.566	0.14551	3.4958	3.64131
Nitrogen 1.3	4	0.658	0.00124	1.566	-0.22532	3.5594	3.33408
Nitrogen 1.4	4	0.658	0.00124	1.566	2.44898	4.0464	6.49538
Nitrogen 1.5	4	0.658	0.00124	1.566	2.44898	4.1722	6.62118
Tin 1.3	8	0.658	0.00995	1.566	-0.01094	4.2814	4.27046
Nitrogen 1.6	4	0.658	0.00124	1.566	2.44898	4.3465	6.79548
Tin 1.4	8	0.658	0.01037	1.566	-0.06178	5.3016	5.23982
Tin 1.5	8	0.658	0.01043	1.566	-0.06178	5.4156	5.35382

Table S 8: Table of electron self energy and cumulant corrections for the fit to the $I\bar{4}3d$ structure

name	ei	third	fourth
Nitrogen 1.1	0	0	0
Nitrogen 1.2	0	0	0
Tin 1.1	5.53846	0	0
Tin 1.2	5.53846	0	0
Nitrogen 1.3	0	0	0
Nitrogen 1.4	0	0	0
Nitrogen 1.5	0	0	0
Tin 1.3	5.53846	0	0
Nitrogen 1.6	0	0.02445	0.0003
Tin 1.4	14.76185	0	0
Tin 1.5	14.76185	0	0

The R-factor by k-weight for this fit is $= 1 \Rightarrow 0.08931$, $2 \Rightarrow 0.06826$, $3 \Rightarrow 0.10772$

When comparing the fits of the R3c structure and the $I\bar{4}3d$ structure, it can clearly be seen that the R3c structure is the more likely structure.

Table S 9: Comparison between fit statistics for $R\bar{3}c$ and $I\bar{4}3d$ structures

Structure	Running R-factor	Reduced χ^2
$R\bar{3}c$	0.0061721	7.5093730
$I\bar{4}3d$	0.0867398	163.4285416

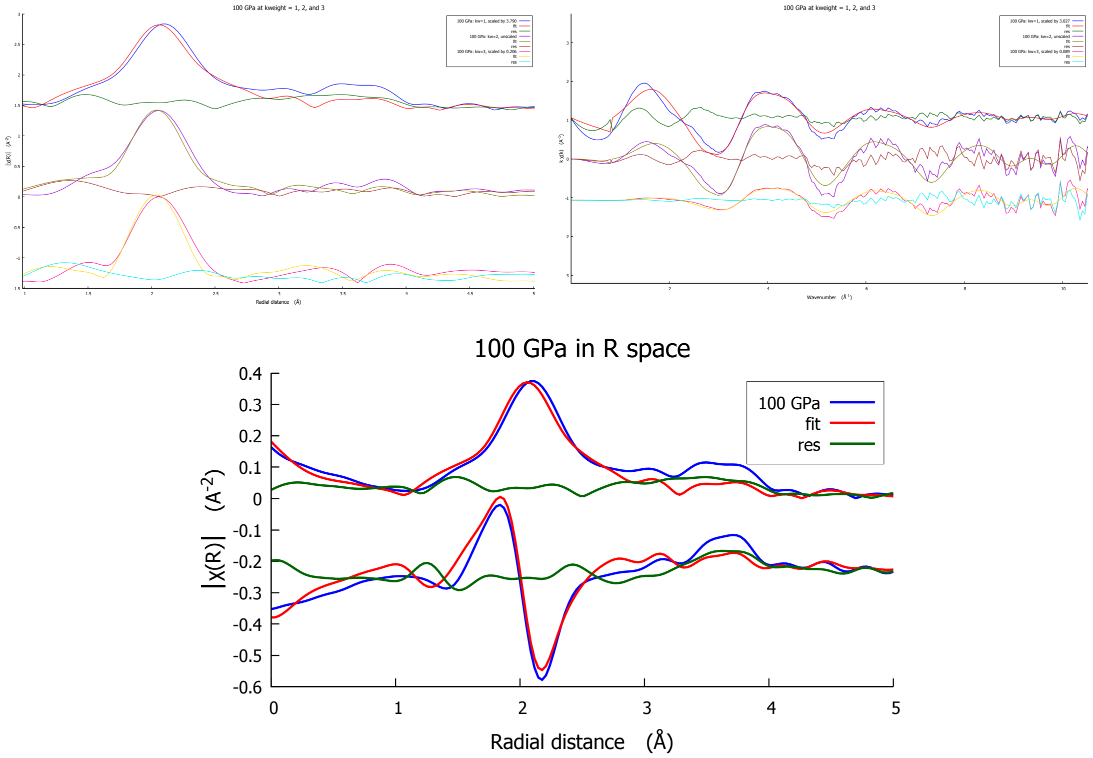

Figure S 6: **Fitting results of 105 GPa data set fit to $I\bar{4}3d$ structure.**
Left Top: Fit results to the magnitude of the fourier transform of the EXAFS spectrum weighted by k, k^2, and k^3. **Right Top:** Fit results to the EXAFS spectrum weighted by k, k^2, and k^3. **bottom:** Fit results with residuals to the magnitude of the fourier transform of the EXAFS spectrum and the real part of the fourier transform of the EXAFS spectrum weighted by k. In the $R\bar{3}c$ structure, the 7 coordinated nitrogen atoms have 4 unique distances from the absorbing tin atom, whereas the $I\bar{4}3d$ structure has 8 coordinated nitrogen atoms with only 2 unique distances from the absorbing tin atom. Though there is one extra nitrogen atom in the $I\bar{4}3d$ structure, they are far more ordered than in the $R\bar{3}c$ structure. Using the more ordered $I\bar{4}3d$ structure type can not properly describe the asymmetry and extra features in the first peak of the EXAFS spectrum. All spectra are phase corrected using the phase amplitude of the first nitrogen shell.

7 Crystallographic information

Table S 10: The unit cell parameters of the three experimentally confirmed phases of Sn_3N_4

Space Group	$Fd\bar{3}m$	$P2_1/c$	$R\bar{3}c$
Pressure	ambient	56 GPa	125 GPa
a (Å)	9.03716(5)	2.976(5)	8.978(7)
b (Å)	9.03716(5)	9.710(9)	8.978(7)
c (Å)	9.03716(5)	5.248(9)	5.12(2)
α, β, γ (degrees)	90, 90, 90	90, 108.13(7), 90	90, 90, 120
V/f.u. (Å^3)	92.258(2)	77.1(2)	59.6 (3)
Fractional coordinates (x, y, z)	Sn (0, 0, 0)<br>Sn (3/8, 3/8, 3/8)<br>N (0.24105, 0.24105, 0.24105)	Sn (0.7156, 0.8481, -0.0316)<br>Sn (0, 0, 0.5)<br>N (-0.06336, 0.18723, 0.31177)<br>N (0.36853, 0.55229, 0.71461)	Sn (0.8120, 0, 1/4)<br>N (0, 0, 0)<br>N (0.5607, 0, 0,25)

8 Electronic structure

Figure 7 shows the electronic band structure of $Fd\bar{3}m$ Sn_3N_4 and the associated partial densities of states. The figure is supporting the claims of the s-character of the disperse conduction band and the p-/d-character of the flat valence band, as stated in the main body of the article.

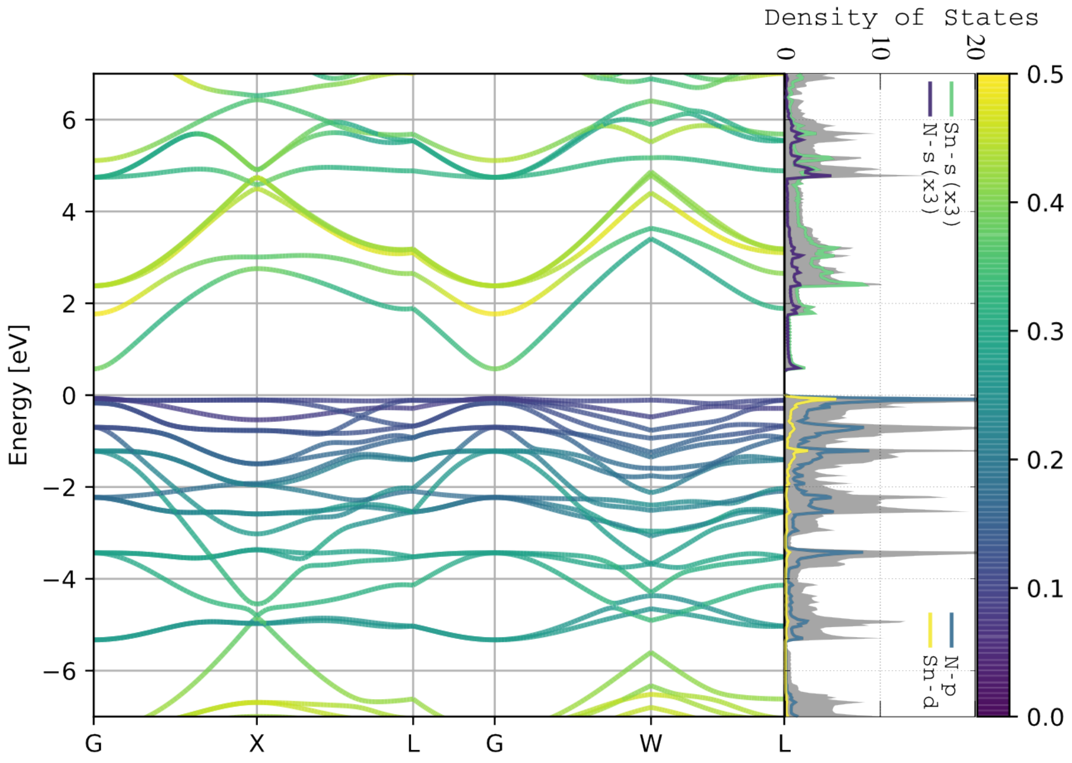

Figure S 7: **Band structure of Sn_3N_4 spinel ($Fd\bar{3}m$) phase along selected high symmetry directions.** Colour map indicates the Sn contributions to the bandstructure, with high values corresponding to high Sn contributions and low values showing mostly nitrogen derived bands. Right panel shows the total density of states of the bands shaded in grey. The partial densities of states for Sn and N associated with different orbitals are shown by colored lines.

9 Bader charge analysis

Bader charge analysis allows for the evaluation of charge localization around specific ions in the system and can help in the identification of the ionization states of atoms. From Figure 8 we see that Sn-atoms are positively ionized, while N-atoms are negatively ionized in the spinel ($Fd\overline{3}m$) phase of Sn_3N_4, congruent with the ionic character of the bonding elucidated in the main body of the article. We observe that with increasing pressure the polarity of the bonding is also increasing, with charges becoming more localized around the respective ions.

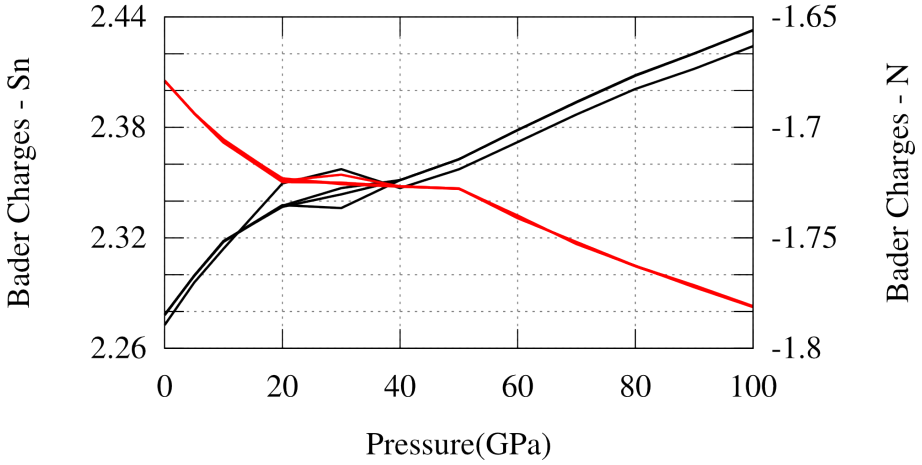

Figure S 8: Bader charges of selected Sn atoms (black lines) and selected N atoms (red lines) in the Sn_3N_4 spinel ($Fd\overline{3}m$) phase as a function of pressure.

10 Charge analysis

The conduction band and valence band charge density for the spinel unit cell is shown in Figure 9 for different pressures. A decrease in the interatomic spacing d from 2.08 Å at ambient to 1.95 Å at 50 GPa and 1.88 Å at 100 GPa is accompanied by an increase in localization of conduction band charge density – most notably around the N atoms and in the interstitial between Sn sites. This increase in charge localization with pressure in turn produces deeper potentials and, as a result, sees the unoccupied levels in the CBM become harder to access energetically, notably this effect is less dramatic in valance charge (bottom panel). Figure 10 shows the changes in the conduction band charge as function of pressure from 40 to 100 GPa. Clearly visible for first neighbors Sn-N atoms (right part), localization increases with pressure. Figure 11 shows the changes in the conduction band charge as function of pressure from 80 to 120 GPa. Despite the electron differences become less pronounced at higher pressures, for the R3c phase substantially gain in ionization potential is seen for first neighbors N-Sn atoms.

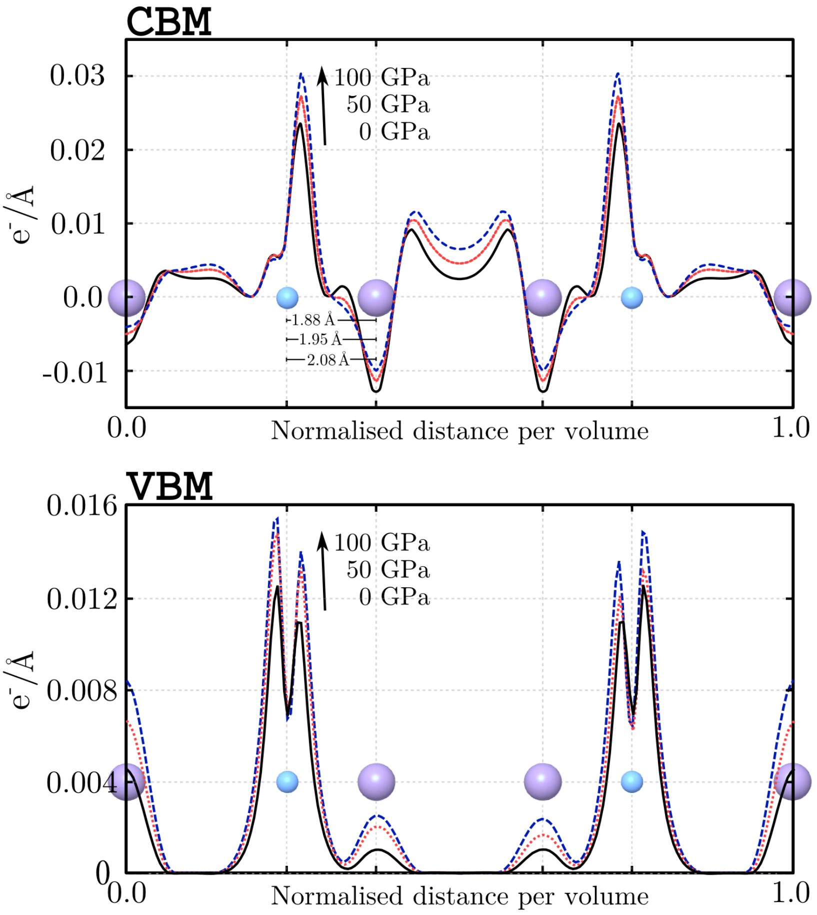

Figure S 9: Changes in charge density conduction band minimum and the valence band for the spinel phase.

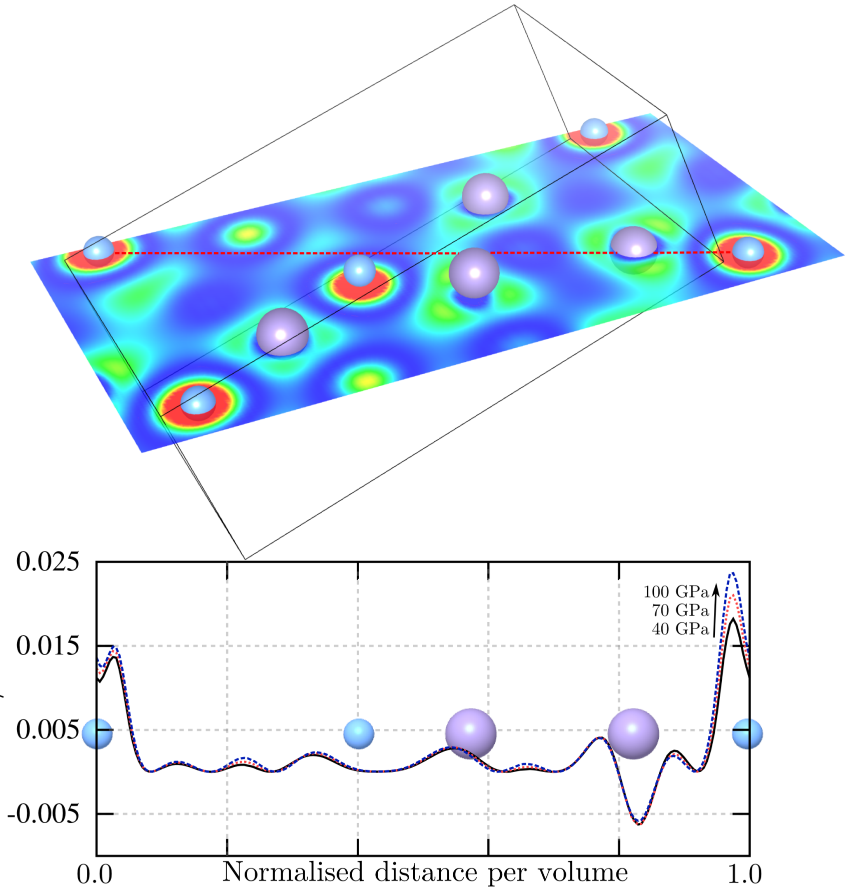

Figure S 10: Bottom panel shows the change in the charge density at the conduction band minimum for a line cut through the plane of the $P2_1/c$ phase identified in the top panel.

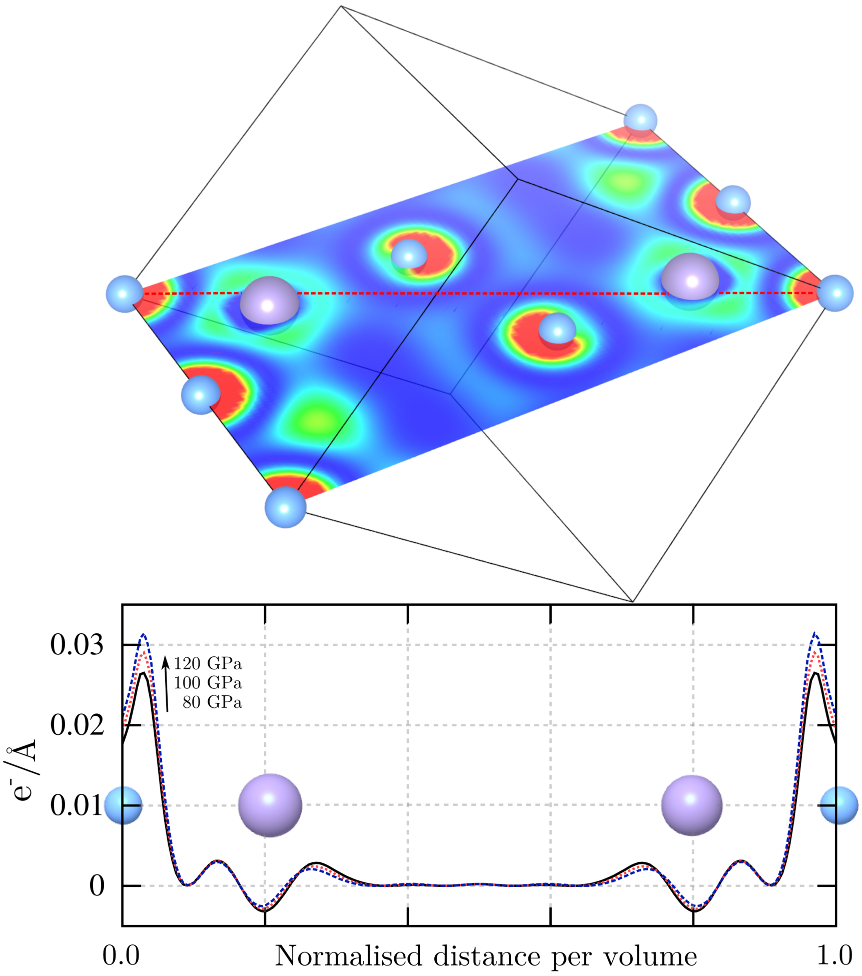

Figure S 11: Bottom panel shows the change in the charge density at the conduction band minimum for a line cut through the plane of the $R\bar{3}c$ phase identified in the top panel.

11 Summary of experimental runs

Table S 11: Summary of the high pressure experiments carried, in sequential time line, out on Sn_3N_4, in the

Gasket	PTM	Pressure range	Heating	XRD	XAS	OBG
Re	–	0 - 120	–	–	–	yes
Re	–	0 - 230	–	–	–	yes
Re	Ne	80 (rapid)	–	yes	–	–
Re	–	0 - 50 W	–	yes	–	–
Re	Ne	0 - 47	–	yes	–	–
Re	Ne	0 - 60	–	yes	–	–
Be	Ne	0 - 64	–	yes	yes	–
Be	KBr	58	CO_2	yes	yes	–
Be	KBr	100	CO_2	yes	yes	–
Re	KBr	60	CO_2	yes	–	–
Re	–	55	Ohmic 250 C Block 400 C	yes	–	yes
Re	–	125	Ohmic 250 C Block 400/600 C	yes	–	yes
none	–	2	-	-	-	yes

12 Optical band gap measurements *via* in-house UV-vis

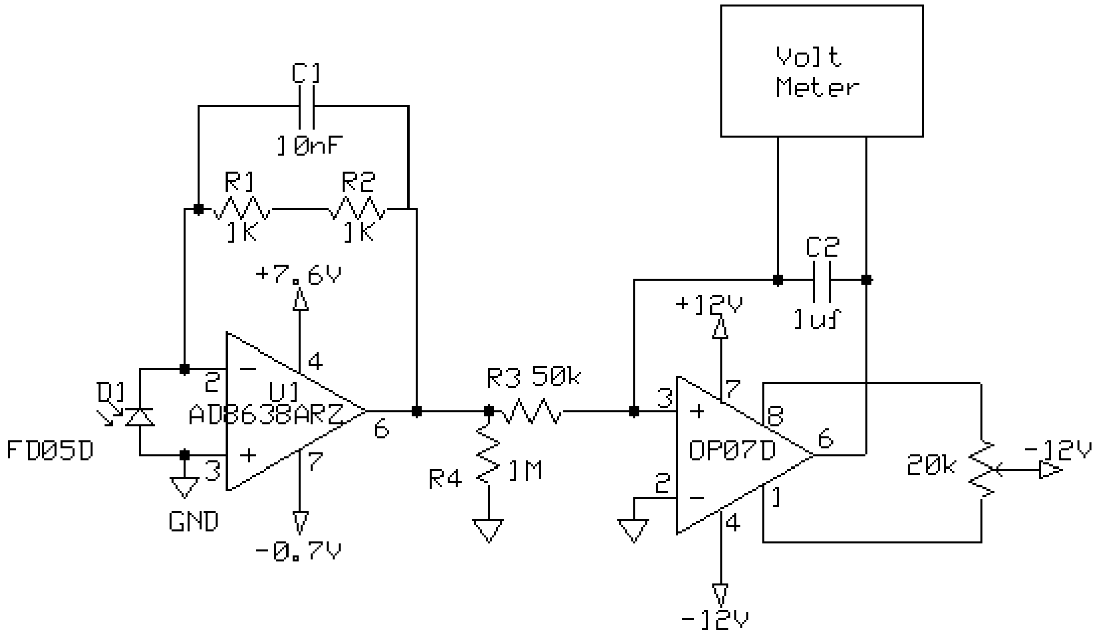

Figure S 12: Circuit diagram for optical absorption measurements, using a Thorlabs FD05P InGaAs photodiode. The diode was attached to an op-amp to minimize any potential reverse bias current. Any signal output by the diode would cause a charge to build up on C2 proportional to the intensity of transmitted light incident on the diode. A voltmeter was then used to read the charge on C2, which was the intensity data point at a given wavelength selected by the monochromator.

Optical absorption measurements were undertaken at UNLV, using our home built design. a 300‘W tungsten bulb was used as the source of polychromatic white light. Two means of data collection were implemented. The first method involved focusing white light through the sample chamber of a DAC and collecting the transmitted light with an Ocean Optics HR2000+ES spectrometer sensitive from 190-1100 nm – allowing for optical measurements from 1.20–3.25 eV. Alternatively, the source light was first sent through a Bauch & Lomb high-intensity monochromator with a transmission diffraction grating with a groove density of 337.5 g/mm. This allowed for the isolation of specific wavelengths in the visible and IR to be sent through a DAC and analysed by a Thorlabs FD05D InGaAs photodiode, with a maximum responsivity at 2300 nm. This allowed for absorption measurements of energies from 0.50–1.70 eV with high efficiency. An integrating circuit was used with the photodiode to allow for long collection times as the monochromator was scanned across a range of wavelengths from the visible into the IR. Fig. 12 is the circuit diagram for this system. The signal from the photodiode would charge a 1 μF capacitor, and a Triplett 1101-b multimeter was used to measure the voltage across the capacitor after a given exposure time. The voltage at each peak wavelength selected by the monochromator allowed for the compilation of a complete absorption spectrum through the sample across the visible and near IR. These measurements

were normalised to I_0 by collecting thought the same, empty, DAC geometry.

References

[1] R. Hrubiak, S. Sinogeikin, E. Rod, and G. Shen, "The laser micro-machining system for diamond anvil cell experiments and general precision machining applications at the high pressure collaborative access team," *Review of Scientific Instruments*, vol. 86, p. 072202, jul 2015.

[2] A. Dewaele, P. Loubeyre, and M. Mezouar, "Equations of state of six metals above 94 gpa," *Physical Review B*, vol. 70, sep 2004.

[3] Y. Akahama and H. Kawamura, "Pressure calibration of diamond anvil raman gauge to 310gpa," *Journal of Applied Physics*, vol. 100, no. 4, p. 043516, 2006.

[4] A. Salamat, R. A. Fischer, R. Briggs, M. I. McMahon, and S. Petitgirard, "In situ synchrotron x-ray diffraction in the laser-heated diamond anvil cell: Melting phenomena and synthesis of new materials," *Coordination Chemistry Reviews*, vol. 277–278, pp. 15–30, Oct. 2014.

[5] S. Petitgirard, A. Salamat, P. Beck, G. Weck, and P. Bouvier, "Strategies for in situ laser heating in the diamond anvil cell at a x-ray diffraction beamline," *J. Synchrotron Rad.*, vol. 21, pp. 89–96, 2014.

[6] C. Prescher and V. B. Prakapenka, "DIOPTAS: a program for reduction of two-dimensional x-ray diffraction data and data exploration," *High Pressure Res.*, vol. 35, pp. 223–230, jun 2015.

[7] C. J. Pickard and R. J. Needs, "High-pressure phases of silane," *Physical Review Letters*, vol. 97, p. 045504, Jul 2006.

[8] C. J. Pickard and R. Needs, "Ab initio random structure searching," *Journal of Physics: Condensed Matter*, vol. 23, no. 5, p. 053201, 2011.

[9] C. Adamo and V. Barone, "Toward reliable density functional methods without adjustable parameters: The PBE0 model," *J. Chem. Phys.*, vol. 110, no. 13, pp. 6158–6170, 1999.

[10] G. Kresse and J. Furthmüller, "Efficiency of ab-initio total energy calculations for metals and semiconductors using a plane-wave basis set," *Comput. Mat. Sci.*, vol. 6, pp. 15–50, 1996.

[11] A. Gulans, S. Kontur, C. Meisenbichler, D. Nabok, P. Pavone, S. Rigamonti, S. Sagmeister, U. Werner, and C. Draxl, "Exciting: a full-potential all-electron package implementing density-functional theory and many-body perturbation theory," *Journal of Physics: Condensed Matter*, vol. 26, no. 36, p. 363202, 2014.

[12] H. Jiang, R. I. Gómez-Abal, X.-Z. Li, C. Meisenbichler, C. Ambrosch-Draxl, and M. Scheffler, "FHI-gap: A code based on the all-electron augmented plane wave method," *Computer Physics Communications*, vol. 184, pp. 348–366, Feb. 2013.

[13] D. Nabok, A. Gulans, and C. Draxl, "Accurate all-electron G_0W_0 quasiparticle energies employing the full-potential augmented plane-wave method," *Phys. Rev. B*, vol. 94, p. 035118, Jul 2016.

[14] B. Mazumder, P. Chirico, and A. L. Hector, "Direct solvothermal synthesis of early transition metal nitrides," *Inorganic Chemistry*, vol. 47, no. 20, pp. 9684–9690, 2008. PMID: 18800827.
